# Surgical Healthcare Workers Knowledge and Attitude on Infection Prevention and Control: A Case of Tamale Teaching Hospital, Ghana

**DOI:** 10.1155/2021/6619768

**Published:** 2021-04-23

**Authors:** Abdul Rauf Alhassan, E. D. Kuugbee, E. M. Der

**Affiliations:** ^1^Department of Surgery, Tamale Teaching Hospital, P.O. Box TL 16, Tamale, Ghana; ^2^Department of Microbiology, School of Medicine and Health Sciences, University for Development Studies, P.O. Box TL 1850, Tamale, Ghana; ^3^Department of Pathology, School of Medicine and Health Sciences, University for Development Studies, P.O. Box TL 1850, Tamale, Ghana

## Abstract

**Background:**

Most morbidities and mortalities related to clinical, diagnostic, and therapeutic procedures are related to infection and the solution to this is good infection prevention and control (IPC) compliance which is influenced by the right knowledge and positive attitude.

**Aim:**

This study aimed to assess infection prevention and control (IPC) knowledge and attitude among healthcare workers at the surgical department of Tamale Teaching Hospital (TTH).

**Methods:**

This study was conducted using a descriptive cross-sectional survey. Data entry and analysis were done using Statistical Package for the Social Sciences (SPSS) version 20 and Graph Pad Prism version 6.05. Tables, frequencies, and percentages were used for descriptive analysis and chi-square analysis for the associations.

**Results:**

Of the 156 participants who responded, 22 (14.1%) were doctors, with 107 (68.6%) nurses, 12 (7.7%) certified registered anesthetics (CRA), and 15 (9.6%) orderlies. Approximately, 50.6% of the respondents were knowledgeable with regard to IPC and 55.1% of the respondents had a good attitude towards IPC. Factors associated with knowledge level were educational level (*p* ≤ 0.001), occupation (*p* ≤ 0.001), marital status (*p*=0.030), and age (*p*=0.030). The occupation was the only factor associated with the attitude level (*p*=0.048).

**Conclusion:**

More than half of the healthcare providers reported good knowledge and attitude towards IPC. Proportionally, more nurses had good IPC knowledge and attitude as compared to other professional groups. Firming up and assimilating universal precaution with routine services by providing training, protocol, rules, and regulation are recommended.

## 1. Introduction

Efficient infection prevention and control (IPC) practices are basic requirements for all health facilities to reduce the morbidity and mortality associated with microbial agents and hence excellent patient outcomes. Healthcare facility-associated infections which are also known as nosocomial infections are acquired during healthcare delivery from patient or healthcare staff or through contaminated equipment, instruments, hands, bed linen, or air droplets [1].

According to World Health Organization (WHO) guidelines on hand hygiene, hand hygiene remains the basic measure proven to be efficient in fighting nosocomial infection, even though its compliance has been very low in both developed and developing countries [2]. And Stilo et al.'s study also indicated that hand hygiene with Marseille soap and with Povi-iodine has the tendency of significantly reducing microbial load and further recommended that hand hygiene be part of the multifaceted strategy of surveillance and control of nosocomial infection [3]. The use of medical equipment such as a stethoscope without disinfection between patients is another source of nosocomial infection. Messina et al.'s study confirmed the possibility of transfer of bacterial from the skin to medical equipment (stethoscope) [4].

The Center for Disease Control and Prevention (CDCP) estimated in 2017 that every year about 1.70 million Americans are affected with hospital-associated infections with all types of microorganisms with some microorganisms difficult to treat with antibiotics [1]. Sub-Saharan African countries have a high incidence rate of hospital-acquired infections ranging from 2.0 to 49.0%. For instance, the prevalence of nosocomial infections in Ghana is reported to be 6.7% [5].

The prevalence of nosocomial infections in TTH has been reported by a previous study to be 8.0%, and this is close to the national point prevalence rate of 8.2%, with surgical site infections being the commonest [6]. According to Kaneko et al., surgical site wound infection accounts for one out of five healthcare-associated infections [7]. Available current literature covering the period 2016 to 2018 has revealed an increase in surgical site wound infections in TTH from 9.3% to 11.5% for overall surgical site infection with 3.4% to 6.0% for deep surgical site infection [8, 9].

An earlier study in 2014, by Apanga et al., recommended further institution-based research such as work practices of healthcare providers to evaluate or identify other factors accounting for the increased surgical site infection in health facilities, particularly in the TTH [10]. To this effect, a recent study by Alhassan et al. on hand hygiene and facemask compliance in TTH among healthcare providers reported a below-average number of participants complying with hand hygiene and a little above seventy percent of them complying with facemask use [11]. Therefore, the main aim of this study was to assess infection prevention and control (IPC) knowledge and attitude among healthcare workers at the surgical department of TTH.

## 2. Materials and Methods

This study was conducted using a descriptive cross-sectional survey among healthcare providers at the surgical department of TTH, Ghana, using a self-designed and self-administrable survey questionnaire using WHO and CDC guidelines on IPC. The kind of questions asked is presented in Tables 1–5. The data collection period was February 2019 to June 2019 and the duration for the study was October 2018 to April 2020. The criteria for inclusion were to be healthcare providers (doctors, nurses, anesthetists, and orderlies) working in the surgical department of the TTH. Excluded were healthcare providers who were not randomly selected, those who denied consent to participate in this study, and all those who have worked less than one month in the surgical department of TTH.

Data entry and analysis were done using Statistical Package for the Social Sciences (SPSS) version 20 and Graph Pad Prism version 6.05. Scores for knowledge and attitude on IPC were done using a sum score for each respondent. The mean score for each section was used to categorize levels of scores for each of these sections (knowledge and attitude) adopting a similar method used in a study by Kassahun and Mekonen as a guide [12]. Descriptive analysis of all variables of the study was done using frequencies and percentages presented using tables. Chi-square analysis was done for association between respondents' demographic characteristics and their IPC knowledge and attitude level. Significance level set for this study was 95%.

Approval to conduct this research in the hospital was gained from the research department of TTH after reviewing the proposal and tool for data collection. Respondents' consented to participate in the study and they were made to know that they had the right to skip any question they feel uncomfortable answering and can withdraw from participating at any time they will. Confidentiality was ensured and any form of harm was avoided.

## 3. Results

A total of 160 questionnaires were administered, of which 156 (97.5%) were filled and returned. The majority (65.4%) of the 156 respondents were males with 34.6% females. The ages of respondents ranged from 21 to 58 years with a mean age of 32.78 ± 6.17 years and a median age of 32.00 years. The modal age group was 30–39 years (58.3%) followed by 20–29 (30.8%). Many (69.9%) of the respondents were married. The years of occupational work experience of respondents ranged between 0.5 and 31 years with a mean of 6.49 ± 5.32 years. The majority (73.7%) of the workers had between 0 and 9 years of working experience followed by 21.8% with 10–19 years of experience. The respondents' years of experience in the surgical department ranged from 0.5 to 25 years with a mean of 3.12 ± 3.00 years. Most (94.9%) of the respondents had between 0 and 9 years of working experience in the surgical department (Table 1).

### 3.1. Respondents' Knowledge of IPC

There were nine items under this section of the questionnaire. A majority (96.8%) of the respondents said they know how to prevent and control hospital-acquired infections. About 78.8% of respondents were, however, familiar with health acquired infection prevention guidelines. On the control of infections, a total of 144 (92.3%) agreed that microbial organisms are not destroyed by using clean water alone, and 142 (91.0%) also agreed that one cannot handle body fluids with bare hands if gloves are not available. More than half (53.8%) of the respondents were not aware of the WHO “five moments of hand hygiene” (Table 2).

All the questions were positively worded with a yes response representing the correct answer and a no representing the incorrect answer. The mean score of all respondents (7.39 ± 1.37) was used as a cut-off point for categorizing the knowledge level. Respondents were classified as knowledgeable (if the respondent scored greater than or equal to the mean score of the correctly answered questions for the whole respondents) or not knowledgeable (if a respondent scored less than the mean score of the correctly answered questions for the whole respondents) (Table 2). Seventy-nine (50.6%) of respondents were knowledgeable with regard to IPC while seventy-seven (49.4%) were not knowledgeable.

### 3.2. The Attitude of Respondents towards IPC

There were seven items under this section of the questionnaire. All the questions were positively worded with a yes response representing the correct answer and a no representing the incorrect answer. The majority (97.4%) of the respondents agreed to wash their hands even if they used gloves (*P* ≤ 0.001). This was followed by 149 (95.5%) respondents who believed that following the prevention guidelines will reduce rates of hospital-acquired infection (*P* ≤ 0.001). A little above half (53.2%) agreed that their workload does not affect the ability to apply infection prevention guidelines (Table 3).

The mean attitude score of all respondents (5.61 ± 2.37) was used as a cut-off point for categorizing attitude level. Attitude levels were classified as a good attitude (if participants scored greater than or equal to the mean score of the correctly answered questions for the whole participants) or poor attitude (if a participant scored less than the mean score of the correctly answered questions for the whole participants). Among all the respondents, 86 (55.1%) had a good attitude and 70 (44.9%) had a poor attitude.

### 3.3. The Bivariate Measure of Association between Respondents' Demography and Knowledge Level on IPC

Pearson Chi-square analysis was done to identify an association between respondent's demographic characteristics and knowledge level. There was significant association between educational level (*p* ≤ 0.001) with medium effect (*ϕ* = 0.32), occupation (*p*=0.001) with medium effect (*ϕ* = 0.33), age group (*p*=0.002) with medium effect (*V* = 281), and marital status (*p*=0.030) with small effect (*ϕ* = 0.17) (Table 4).

### 3.4. The Bivariate Measure of Association between Respondents' Demography and Attitude towards IPC

The Chi-square analysis of attitude level of respondents and respondents' demographic characteristics identified evidence of only respondents' occupation to be associated with attitude level, *p*=0.048 with medium effect *V* = 0.23 (Table 5).

## 4. Discussion

The current study conducted at the surgical ward of the TTH found the respondents to be young with a mean age of 32.78 ± 6.17 years; many were males. Again, the majority were married. This differs from two previous studies conducted in southern Ghana where most of the participants were females [13, 14]. For instance, a study by Hayeh, at the La General Hospital in Accra, found 71.4% of their study population to be females [14]. The majority of the respondents had tertiary education and this is in line with Kondor's study where the majority of the respondents' also had tertiary education [13].

In this study, most of the respondents identified the hospital as the main source of nosocomial infection. They also agreed that all staff and patients should be considered potentially infectious regardless of their diagnosis and knew how to prevent and control hospital-acquired infections. This is in line with Stubblefield's study, that to confirm infection as nosocomial, the source of infection must be from the hospital [15].

Again the great majority agreed nosocomial infection can be transmitted by medical equipment such as syringes, needles, catheters, stethoscope, and thermometers, and those microbe organisms are not destroyed by using clean water alone. This supports a study by Al-Khalidi, that nosocomial infections are acquired during healthcare delivery from patient or healthcare staff or through contaminated equipment, instruments, hands, bed linen, or air droplets [1].

In this study, more than ninety percent of the respondents knew that you cannot handle body fluids with bare hands if gloves are not available. The study found that 48.1% of the study population did not have an idea with regard to the presence or absence of an infection control team in the hospital. Furthermore, 78.8% were familiar with hospital-acquired infection prevention guidelines. This is lower as compared to Mukwato et al.'s study which indicated 86.0% of respondents have heard of infection prevention guidelines [16].

Less than the average of the respondents knew about the WHO's 5 moments of hand hygiene. According to Mathur, the most efficient, easiest, and least-cost method of infection prevention in a healthcare setting is hand hygiene [17]. And the five moments of hand hygiene by WHO is a proven tested approach, which is reasonable and user-friendly for hand hygiene in all healthcare settings that all healthcare workers must know [18].

Approximately, 50.6% of the respondents were knowledgeable. This value is lower than the findings published in previous studies in Ghana and other West African Countries [13, 19, 20]. For instance, Kondor, a study on IPC conducted in La General Hospital in Accra Ghana, found that the great majority (97.0%) of the participants were knowledgeable [13]. Similarly, Iliyasu et al., in their study on knowledge and practices of infection control among healthcare workers in a Tertiary Referral Center in North-Western Nigeria, reported an overall high median knowledge of 70.0% [19]. A study by Cawich et al. identified 81% of staff with knowledge of infection control practices against 41% of them with compliance with IPC [21]. However, the proportion of the participants in the Tamale study who were knowledgeable was higher than the 20.3% reported in a Trinidad study [22]. Going by the KAP model by Bano et al., healthcare providers in TTH are more likely to comply with IPC as compared to those in Trinidad et al. [22, 23].

In this study, respondents' occupation was associated with IPC knowledge level; nurses had the highest number of knowledgeable workers with regard to IPC, followed by doctors, and then anesthetists, and all the participating orderlies scored below the average IPC knowledge score of all the respondents. This result was quite different from similar studies in Ghana and Nigeria, where a good proportion of doctors had good knowledge, followed by laboratory people, then nurses, and finally orderlies [24, 25].

Also, there was a significant association between educational level and respondents' IPC knowledge level. This is similar to a study in Ethiopia, which had an education status association with IPC knowledge [26]. This, however, differs from a study that found no significant association between respondents' education level and IPC knowledge level [27].

The study also found a significant association between respondents' marital status and their IPC knowledge level. The majority of those married were not knowledgeable and the majority of those being single were knowledgeable. This is in line with Desta et al.'s (2018) study which indicated an association between marital status and IPC knowledge level [26].

Among the study variables for attitude towards IPC, the following had the most correct response; the majority of the respondents agreed to have to wash hands even after gloves use. Most of them agreed that policies and procedures for infection control should be adhered to at all times. More than ninety percent agreed to attend in-service training/workshops related to infection prevention and control regularly. This is required because Desta et al. identified a significant association between in-service training and IPC practice [26].

The great majority of the respondent believed following infection prevention guidelines will reduce nosocomial infection as Desta et al. identified a significant association between adherence to infection prevention guidelines and IPC practice [26]. Similarly, most of the respondents agreed that it is their responsibility to comply with the IPC guidelines and procedure guidelines of their unit. Healthcare providers must comply with IPC guidelines [28].

The least performed attitude variable believed that the workload affects their ability to apply infection prevention guidelines. This differs from the findings of Kondor's study who reported that time constraint contributed 66.4% to noncompliance towards IPC [13]. Healthcare facility bed occupancy exceeding the standard capacity of the health facility is associated with increased risk of nosocomial infection and this is complicated with inadequate healthcare providers [18].

About 55.1% of them had a good attitude and 44.9% had a poor attitude. There is a need for improvement since one of the strongest pillars of IPC compliance is a positive or good attitude towards IPC [29]. This current study finding is in line with similar earlier studies [22, 29–31].

A study on assessment of knowledge, attitude, and practice of healthcare workers on infection prevention in a health institution in Bahir Dar city administration showed attitude score of 55.6% translated to almost the same practice of 54.2% [29]. A study by Unakal et al., in three hospitals in Trinidad and Tobago, indicated an attitude level of 53.3% which is translated to a practice level of 56.0% for infection prevention and control, a sign that attitude influences the practice [22].

The occupation was associated with attitude level; the majority of nurses had a good attitude towards IPC, followed by doctors, then orderlies, and lastly the anesthetists. This is a similar result as compared to a study by McGaw et al. (2012) in West Indies, Jamaica, which indicates an overall higher attitude (*p*=0.001) towards IPC by nurses than doctors [32].

This study is not without limitation since not all workers in the department were included in this study but a significant sample of the total population.

All healthcare providers were not included in the study due to limitations of resources such as time and money. Therefore, the study relied on a sampling of the population for the study. The sample size for this study was determined using Krejcie and Morgan (1970) sample size determination table [33]. With a known population of 245, a sample size of 160 was used for this study. A stratified random sampling method was used to divide the study population into strata according to their profession and simple random sampling used to select respondents from each stratum proportionally to their population.

## 5. Conclusion

The aim of this study to assess IPC knowledge and attitude among healthcare workers was achieved with survey design. More than half of the healthcare providers reported good knowledge and attitude towards IPC. Proportionally, more nurses had good IPC knowledge and attitude as compared to other professional groups. Firming up and assimilating universal precaution with routine services by providing training, protocol, rules, and regulation are recommended.

## Figures and Tables

**Table 1:** Sociodemographic characteristic of study respondents.

		Frequency (*n* = 156)	Percent (100%)
Sex	Male	102	65.4
	Female	54	34.6

Age group	20–29	48	30.8
	30–39	91	58.3
	40–49	12	7.7
	50–59	5	3.2

Marital status	Married	109	69.9
	Single	47	30.1

Education level	Primary	6	3.9
	Secondary	8	5.1
	Tertiary	142	91.0

Occupation	Doctor	22	14.1
	Nurse	107	68.6
	Anesthetics'	12	7.7
	Orderly	15	9.6

Duration of work	0–9	115	73.7
	10–19	34	21.8
	20–29	5	3.2
	30–39	2	1.3

Duration of work in the surgical department	0–9	148	94.9
	10–19	7	4.5
	20–29	1	.6

Source: field survey, 2019.

**Table 2:** Respondents' response on IPC knowledge.

Item or question	Correct response	Frequency (*n* = 156)	Percentage
Sources of surgical site wound infections			
Hospital is a source of nosocomial infection	Yes	151	96.8
Nosocomial infection can be transmitted by medical equipment such as syringes, needles, catheters, stethoscopes, thermometers, etc.	Yes	144	92.3
All staffs and patients should be considered potentially infectious regardless of their diagnosis	Yes	151	96.8

Knowledge of surgical site wound infection preventive methods			
Do you know how to prevent and control hospital-acquired infections?	Yes	151	96.8
Are you familiar with hospital-acquired infection prevention guidelines?	Yes	123	78.8
There is no infection control team in the hospital	Yes	75	48.1
Microbe organisms are not destroyed by using clean water alone	Yes	144	92.3
Do you know WHO's 5 moments of hand hygiene?	Yes	72	46.2
You cannot handle body fluids with bare hands if gloves are not available	Yes	142	91.0

Source: field survey, 2019.

**Table 3:** Respondents' response on attitude towards IPC.

Item or question	Correct response	Frequency (*n* = 156)	Percentage (%)
I have to wash my hands even if i used gloves	Agree	152	97.4
Policies and procedures for infection control should be adhered to at all times	Agree	151	96.8
I should attend in-service training/workshop related to infection prevention and control regularly	Agree	150	96.2
The workload does not affect my ability to apply infection prevention guidelines	Agree	83	53.2
It is my responsibility to comply with the hospital-acquired infection guidelines	Agree	145	92.9
I believe that following the prevention guidelines will reduce rates of hospital-acquired infection	—	149	95.5
I have to follow the procedural guidelines of the unit	Agree	145	92.9

Source: field survey, 2019.

**Table 4:** Chi-square analysis of the association between respondents' demography and knowledge level on IPC.

		IPC knowledge level		Total	*X* ^2^	df	*P* values	Phi (*ϕ*)/Cramer's *V*
		Not knowledgeable	Knowledgeable					
Sex	Male	51	51	102	0.048^a^	1	0.826	0.018
	Female	26	28	54				
Total		77	79	156				

Marital status	Married	60	49	109	4.681^a^	1	0.030	0.173
	Single	17	30	47				
Total		77	79	156				

Age group	20–29	14	34	48	12.336	2	0.002	0.281
	30–39	55	36	91				
	40–59	8	9	17				
Total		77	79	156				

Educational level	Lower	14	0	14	15.780^a^	1	≤0.001	0.318
	Higher	63	79	142				
Total		77	79	156				

Occupation	Doctor	10	12	22	17.262^a^	3	0.001	0.333
	Nurse	46	61	107				
	Anesthetics	6	6	12				
	Orderly	15	0	15				
Total		77	79	156				

Source: field survey, 2019.

**Table 5:** Chi-square analysis association between respondents' demography and attitude towards IPC.

		IPC attitude level		Total	*X* ^2^	df	*p* values	Phi/Cramer's *V*
		Poor	Good					
Sex	Male	51	51	102	3.133^a^	1	0.077	0.142
	Female	19	35	54				
Total		70	86	156				

Marital status	Married	46	63	109	1.043^a^	1	0.307	−0.082
	Single	24	23	47				0.082
Total		70	86	156				—

Age group	20–29	20	28	48	1.568	2	0.457	0.100
	30–39	40	51	91				
	40–59	10	7	17				
Total		70	86	156				

Educational level	Lower	8	6	14	0.936^a^	1	0.333	0.077
	Higher	62	80	142				
Total		70	86	156				

Occupation	Doctor	13	9	22	7.916^a^	3	0.048	0.225
	Nurse	40	67	107				
	Anesthetics'	8	4	12				
	Orderly	9	6	15				
Total		70	86	156				

Source: field survey, 2019.

## Data Availability

All data related to the findings of this study are available from the corresponding author upon request.
